# It's hard to forget: resetting memory in delay-match-to-multiple-image tasks

**DOI:** 10.3389/fnhum.2013.00765

**Published:** 2013-11-14

**Authors:** Volodya Yakovlev, Yali Amit, Shaul Hochstein

**Affiliations:** ^1^Neurobiology Department, Life Sciences Institute and Safra Center for Brain Research, Safra Campus, Hebrew UniversityJerusalem, Israel; ^2^Departments of Statistics and Computer Science, Chicago UniversityChicago, IL, USA

**Keywords:** memory, forgetting, familiarity, recognition, multiple memories, working memory, reward effects

## Abstract

The Delay-Match-to-Sample (DMS) task has been used in countless studies of memory, undergoing numerous modifications, making the task more and more challenging to participants. The physiological correlate of memory is modified neural activity during the cue-to-match delay period reflecting reverberating attractor activity in multiple interconnected cells. DMS tasks may use a fixed set of well-practiced stimulus images—allowing for creation of attractors—or unlimited novel images, for which no attractor exists. Using well-learned stimuli requires that participants determine if a remembered image was seen in the same or a preceding trial, only responding to the former. Thus, trial-to-trial transitions must include a “reset” mechanism to mark old images as such. We test two groups of monkeys on a delay-match-to-multiple-images task, one with well-trained and one with novel images. Only the first developed a reset mechanism. We then switched tasks between the groups. We find that introducing fixed images initiates development of reset, and once established, switching to novel images does not disable its use. Without reset, memory decays slowly, leaving ~40% recognizable after a minute. Here, presence of reward further enhances memory of previously-seen images.

## Introduction

The field of memory is the subject of intense attention and research effort, with thousands of papers devoted to this important, exciting field. Particular attention is paid to understanding neural substrates of memory and modeling memory mechanisms with physiologically realistic components and behavioral capacities. Nevertheless, relatively little effort has gone into study of the complementary process of required forgetting. Some studies may have analyzed forgetting as not remembering, but we are interested more in active forgetting, in cases where it is better to forget; (see below, Summary and Conclusions, for studies of directed forgetting). Any step towards, understanding how we forget would help us understand memory, at least constraining possible memory models. A breakthrough in understanding forgetting may also be of great clinical significance. A central goal of the current study was to study primate forgetting in performance of a task which was specifically devised so that good performance requires a level of forgetting, and comparing it to performance of a comparable task which does not. We ask how efficient is forgetting, and how effective when not required at all.

**Figure 1:**
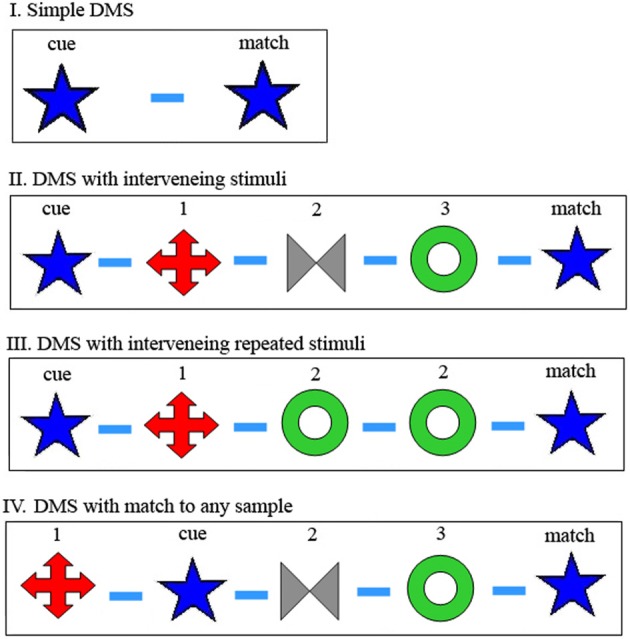
**Modifications of Delay-Match-to-Sample (DMS) task. (I)** In the original DMS task, the first stimulus is the cue and the second is either a match or a non-match to the cue. Participant remembers the cue and responds differently for match or non-match. **(II)** DMS with multiple samples again requires memory of the first, cue stimulus and waiting for its match, which may appear following multiple non-match stimuli. **(III)** Same as **(II)**, but there may appear matches to non-cue (not first) stimuli, which should be ignored. **(IV)** DMS with multiple samples, any one of which may be the cue. Participant remembers all previous stimuli of the trial, and responds to a match to any one of them. In the examples shown for **(II–IV)**, there are three samples presented besides the cue and match, but this number is variable and unknown to the participant. Symbols schematically represent images presented as sample, cue and match. Dash represents delay between image presentations, in our case always 1 s.

The Delay-Match-to-Sample (DMS) task has been used in countless studies of human and animal memory (Ferster, 1960). The task has gone though a number of modifications, making the task more and more challenging to the participant, as demonstrated in Figure 1. The physiological correlate of memory of the cue during the delay period or periods before presentation of the match stimulus, called delay activity, is an elevated (or perhaps diminished) level of neural activity following stimulus presentation (Fuster and Alexander, 1971; Kubota and Niki, 1971; Fuster and Jervey, 1981; Goldman-Rakic, 1995; Wang, 2001). It is assumed that delay activity recorded in one neuron reflects such activity in multiple interconnected cells and that enhanced synaptic interconnectivity in this set underlies long-lasting reverberating delay activity, called attractor activity (Amit, 1995; Amit and Brunel, 1997). It was found that a stimulus must be presented many times (~100 times) to develop delay activity for that stimulus (Miyashita and Chang, 1988; Erickson and Desimone, 1999), presumably by strengthening synapses among neurons in the set. Thus, delay period attractor activity reflects an image being in active Working Memory, while the underlying strengthening of the synapses reflects more permanent long-term memory. This long-term memory synaptic substrate is essential for enabling initiation and maintenance of the Working Memory neuronal activity (Amit, 1995; Amit and Brunel, 1997). In a separate accompanying paper we describe an attractor network model that can accommodate these different tasks (by properly modulating noise and inhibition), and that employs simple mechanisms for readout of the match and for memory reset (Amit et al., 2013).

A common feature of experimentation with the variety of DMS tasks has been use of a fixed set of well-practiced images as visual stimuli. This allows acquaintance with the stimuli, as required to build up delay activity. But this advantage comes at a cost: Using well-learned stimuli presents a challenge for the participant who must determine if the remembered image was seen in the same trial or in a preceding trial. Only a match to an image seen in the same trial should trigger a response. Thus, memory must include the trial number, or, equivalently, at the transition from one trial to the next, all “old” images must be marked as such: There needs to be some sort of “reset” mechanism between trials (Yakovlev et al., 2008).

Assuming the delay activity attractor model, reset may be seen as turning off the delay activity between trials. An important characteristic of such a reset for these tasks is that it will turn off the delay activity Working Memory of any of the images seen up to now, so that seeing them again, in a following trial, will not trigger a motor response. At the same time, such a reset would not destroy the long-term memory synaptic structure acquired through the learning process. The images would still be able to initiate Working Memory activity when presented as a sample in following trials.

Thus, accepting the reverberating delay activity model, it is easy to perceive reset as turning off this reverberation. In this way, assuming match recognition involves comparison of active reverberations and stimulus-initiated activity, only delay activity which derives from images seen in the current trial will initiate recognition of a match image. For DMS tasks I, II, and III in Figure 1, the cue image must initiate delay activity corresponding to this cue image for successful identification of the match. For task III it is essential that subsequent images are not entered into Working Memory in the same way, whereas for task II (at least as it is traditionally reported in the literature) it is immaterial if subsequent images enter Working Memory since they are never tested. However, for DMS task IV, the participant does not know in advance which sample will be the cue image, so that every sample must initiate an (independent) delay activity attractor, representing the corresponding presented image, so that the match stimulus will be recognized as such. All these sets of activity need to be reset, i.e., turned off, at trial end.

As mentioned above, we introduced a new task type (Figure 1, DMS task IV) where all the images seen must be remembered, since any can act as cue to be matched by the final stimulus (Yakovlev et al., 2008). Furthermore, in addition to trials with a small fixed set of well-trained, often seen images, we introduced trials with an unlimited set of novel images. That is, we used new stimulus images on every trial, images that the participant had never seen before. There are two major predictions that may be expected from this difference between tasks with a fixed set vs. novel images: First of all, since the images in the novel set were not viewed many times, there should not have been sufficient opportunity to set up enhanced synaptic connections and therefore, no reverberating activity. Thus, accepting the reverberating attractor model, one might expect that it would be impossible to perform this task with novel images. (Note that novel images were not used even in recent reports of persistent activity in some prefrontal neurons of monkeys not performing a memory task (Meyer et al., 2007). Also, this activity was turned off by subsequent presentation of a different image, so is irrelevant to our multi-image memory task.) Secondly, since the images are all novel, and images from previous trials will never appear again, there should be no need for a reset mechanism.

However, previous studies have shown that it is easy to perform DMS tasks with large sets of images or even novel images. In fact, performance is actually better with larger sets. In the study of Mishkin and Delacour (1975 see also Eacott et al., 1994), each trial had two parts, a pre-trial with the sample alone, and a test with the sample and another stimulus; the monkey was trained to remember which was this trial's sample. They found very poor performance when using a single pair of stimuli for all trials, and far superior performance when using novel stimuli. They concluded that it is easier to discriminate familiarity from novelty than to remember which familiar stimulus was this trial's sample. One experiment by Sands and Wright (1980; see also Basile and Hampton, 2010) had monkeys report if a probe item was among three preceding samples. Performance was far superior for sample chosen from a pool of 200 items vs. only 6 items, suggesting proactive interference was a major problem.

We previously tested macaque monkeys with DMS task IV, which we call the Delay Match to Multiple Sample (DMMS) task, first with a set of 16 fixed images, presented consistently but in random order, and then with an infinite set of novel images (Yakovlev et al., 2008). Despite the predicted lack of delay activity, the monkeys were able to perform the task with novel images and performance with novel images was better than with the fixed set of well-practiced images. We explained this finding by suggesting that rather than depending on delay activity, performance relied on simply judging whether the image were ever seen before—familiarity memory instead of recognition memory. This was modeled by having the cue leave a weak synaptic trace, insufficient to trigger reverberating activity, but sufficient to create a differential response during match presentation (Yakovlev et al., 2008). In an accompanying paper, we show that this can be seamlessly integrated into the attractor network model for Working Memory (Amit et al., 2013).

The second prediction, that with novel images there should be no need for a reset mechanism, was also not entirely borne out: We introduced a small number of catch trials with images from previous trials, and found only a slightly larger number of false positive responses here than with the fixed set of images. That is, there was a reset mechanism used for the infinite set, as well. It may be that a new reset mechanism was set up, or that the reset mechanism acquired during performance of the fixed-set task was transferred to the novel-set task and still used, though somewhat less effectively, during performance of the subsequent novel set task. Presumably this reset mechanism is not the turning off of reverberating delay activity, since such activity is assumed to be absent for novel stimuli.

These findings bring up a number of essential questions regarding the nature of the reset mechanism. If reset is simply turning off attractor reverberations, it should not transfer to familiarity memory of novel images. Therefore, having found reset for novel images, suggests there may be two independent reset mechanisms, which are either inherent or rapidly learned. If so, we should find few False Positives (FPs) also for monkeys not trained on a fixed set of images before testing with novel images. The alternative is that reset must it be acquired or learned, and that it is transferred from fixed set training to novel set test. Another possibility is that the monkeys in the above-described experiment may have acquired a new reset mechanism for the novel-images task, and that this acquisition is independent or depends on prior experience with the reset used for the fixed-set task. If reset for novel images is innate or can be learned very rapidly and does not require prior experience with reset for fixed images, we might expect that inexperienced monkeys tested with novel images will not have many FPs. But, if reset is learned slowly, and/or requires transfer or at least experience with the fixed, then we may expect that inexperienced monkeys, tested with novel images without prior experience with a fixed set, would not have a reset mechanism. This should show up with a large number of False Positive (FP) responses on catch trials. To determine which of these hypotheses is to be accepted and which rejected, we now trained two new inexperienced monkeys on the *novel* images task. Will they have a reset mechanism, inherently or acquired gradually during training?

## Methods and procedures

Two groups of inexperienced Macaca fascicularis monkeys learned to perform a multiple image memory task, as shown in Figure 1IV, i.e., our extended DMS task (Yakovlev et al., 2005, 2008), where samples are sequences of images. Trial protocol is shown in Figure 1IV, a modified, delayed-match-to multiple-sample (DMMS) task (Yakovlev et al., 2005). A sequence of images of random length (with two to seven images) was presented and participants reported whether any (test) image was a repetition of any one of the previous images in the sequence. Thus, on each trial, they were shown 1–6 sample images (*n*) followed by a Match—a repetition of one of the earlier images, which is called the Cue. The task was to recognize and respond to Cue—Match repetition. We randomized sequence length, Cue position (*q*, from start of trial), and images presented, so that perfect performance required holding in memory all of the sample images of the current trial.

Each trial began when the monkey pressed a response bar, following presentation of a slowly flickering small fixation point at the screen center. The fixation point continued alone for 1 s followed by a series of 1 s image presentation and 1 s inter-stimulus-interval until either the monkey released the bar or the presented image was a match to which the monkey released the bar or failed to (during its presentation or in the 0.5 s thereafter). Correct bar release was rewarded with a drop of apple juice (Hit); failure to release was considered a Miss; premature bar release is a FP. Each of these three ended the trial. Inter-trial-interval was 2 s for correct trials or 4 s following a Miss or FP response. During the inter-trial-interval the flickering fixation point was turned off. A bar press during this interval prolonged it for an additional 2 s.

In one variant, the images of the sequence of every trial were selected from a limited fixed set of 16 images, so that during training every image had been seen hundreds of times. In the other variant, sequences were taken from an “infinite” set, so that each image was used only once, except when it appeared a second time in the same trial as a Cue-Match repeat, or when planted in “catch” trials, to study FPs; see below. Any image in the sequence of a trial (except the last) can be the Cue which will be repeated at the end of the trial, and any image (but the first) can be this final Match. When an image is repeated (e.g., A, B, C, C or A, B, A) the subject is supposed to respond (and receives a juice reward for correct response). Images were multicolored drawings, clip art images of patterns, photographs, or cartoon-like drawings of objects (Art Explosion), each of 200 × 200 pixels. One group was first trained and tested on a limited fixed set of images and only later tested with an “infinite” set. We shall call this group the Fixed Set Trained or FST group. The second group had no experience with limited fixed sets before training and testing with an “infinite” set, and later was tested—for the first time—with a limited fixed set. We call this group the Novel Set Trained or NST group. Further details have been presented previously (Yakovlev et al., 2005, 2008). Animal behavior experimentation was performed according to National Institutes of Health and Hebrew University guidelines.

**Figure 2:**
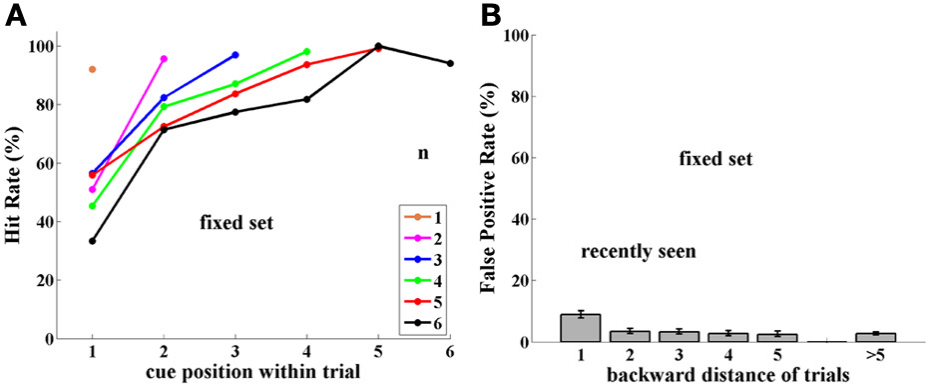
**Experiment I, Group FST (Fixed Set Trained), tested with a fixed set of 16 images. (A)** Performance (Hit rate %), as a function of cue position *q*, and thus, distance from the match stimulus, which is always the last of the trial, for each trial length (color-coded number of samples, *n* = 1,6). **(B)** False Positive rate (FP %), tested as a function of number of trials back to prior appearance of image. Note somewhat higher FP rate for 1-back repetitions. Error bars are Confidence Intervals, which were ± 2–10%, depending on sample size; see Supplementary Material.

**Figure 3:**
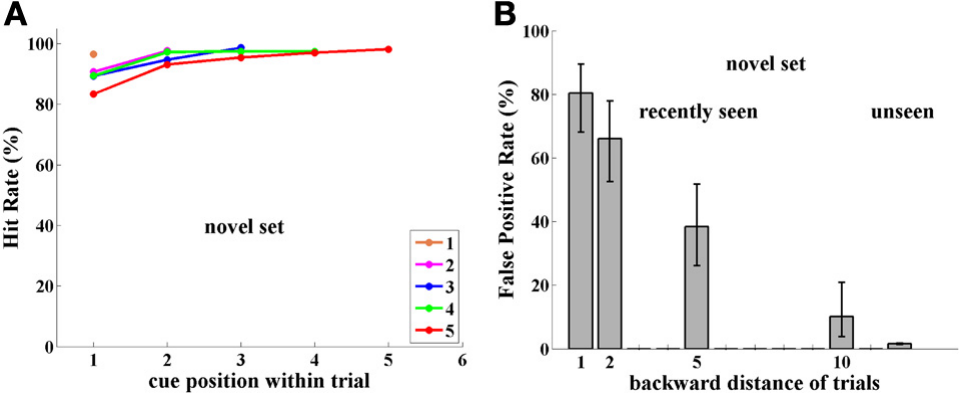
**Experiment I, Group NST (Novel Set Trained), tested with novel images. (A)** Performance (Hit Rate %); note performance is high and independent of cue position, unlike results in Figure 2A for the FST group tested with a fixed image set. **(B)** FP rate; note high FP rate compared to Figure 2B for FST group tested with a fixed image set.

Statistical tests for performance Hit and FP rates was based on the data of Figures 2, 3, 5–7, (as presented in Tables in the Supplemental Material). The Hit rate is Hits/(Hits plus Misses) and the FP rate is FPs/(FPs plus Correct Rejections). Confidence Intervals were calculated using Tim Ross' Accurate Confidence Interval Matlab tool. Significance is judged using the binomial test for proportions and a pooled two-sample *p*-test. Data for all sessions of the two monkeys in each group were pooled for statistical analysis, but we looked at individual participant data as well and results are the same and significant for each one alone. In general the number of trials for each condition was determined by the need for a sufficient number of “catch” trials for the novel images task, while keeping their rate and number low enough that they not be noticed and learned.

## Results

### Experiment I

In Experiment I, monkey behavior for the DMMS task was tested with the same image sets that were used during training. The testing procedure started when performance was stable and at least 80% correct.

#### Group FST

***Performance.*** Monkeys B and T were tested for 4,616 trials with a fixed set of 16 images, which had been heavily learned by them. Performance (Hits relative to Hits plus Misses) strongly depended on trial length, as well as on position within the trial of the cue (the sample repeated by the match stimulus). Thus, when cue and match were adjacent (cue position = number of samples), performance was close to 90%; however, when a few stimuli intervened between cue and match stimuli, performance strongly decreased. This is shown in Figure 2A. (For all figures, data are tabulated with their Confidence Intervals in the Supplementary Material).

***False positives.*** Using a limited set of images leads to a large probability of the same image being repeated in close trials. If an image is preserved in memory, then when it appears again in another trial, as a sample, this repetition may confuse the monkey, which may consider it a match repetition, evoking a response, which is a FP error. Indeed, this is what occurred in numerous cases, but only for images shown in adjacent trials, i.e., 1-back from the current trial (9% FPs). When repeated images were shown further than 1-back, however, the FP error rate was lower, and independent of how far back they were from the current trial. This is demonstrated in Figure 2B, where FP rate is the ratio of FP relative to FP plus Correct Rejections—for each distance.

#### Group NST

***Performance.*** Monkeys D and L were trained on the same paradigm, but with an unlimited set of images. That is, in every trial they were presented with novel, never before seen images. One image in the current trial was randomly chosen to be repeated as a match—the only case where an image was presented twice. Learning this paradigm was somewhat slower than that for the preceding method with a fixed set of trained images.

Surprisingly, performance of monkeys in Group NST (4,751 trials) was 90% or more and was significantly better than that for monkeys who were tested with a fixed set of images (two-sample pooled *p*-test; *p* < 0.001, for group and for each monkey separately). Indeed performance was significantly better for all cases (*p* < 0.05), except when the cue immediately preceded the match stimulus, as demonstrated in Figure 3A. There is virtually no dependence on cue position, i.e., on distance of the match from the cue, as was found to be so strong for performance with the fixed set of images; (compare Figures 2A, 3A). Here, performance is near ceiling for all cue-to-match distances, which may hide some cue-distance dependence. The only exception was for cue position 1, that is, when the cue was the very first sample at the beginning of the trial. Here, performance was somewhat poorer, for every trial length (and cue-match distance). This may be due to an interaction between the representations in infero-temporal (IT) and pre-frontal (pF) cortex, as described in the Discussion. Note that the first image is special in that it can never be the reward-producing match stimulus.

***False positives.*** Since monkeys D and L were tested with novel images, they were never presented with a repetition of images from previous trials. This was strictly adhered to during training, as the monkeys learned the task. However, in order to estimate how novel images persist in memory through the sequence of trials, during testing we introduced “catch trials,” as follows: in each testing session we intentionally repeated one randomly chosen image from 1, 2, 5, and 10 trials back. Thus, in each session only four catch trials were presented, so that fewer than 5% of trials contained a catch image. This experiment was conducted over 30 sessions for each monkey, so that catch images were shown 30 times for each back distance, for each monkey, (though, of course, not the same ones). This stringent limitation was set so that the monkeys would not learn to expect such trials.

The “catch image” was used only as a sample and was never repeated at the end of the test trial as a match. However, it could have been the cue and match in its previous *n*-back trial. In such conditions, any image may potentially be shown a maximum of three times: cue and match in a previous trial, and sample image in the “catch trial.”

The results for monkeys D and L catch-trial FPs reveal a surprising ability for Novel images used in this experiment to survive through the sequence of trials. Images seen 1-trial-back provoked 80% FPs, 2-trials-back, 66%, and 5-trials-back, 38%. Even 10-trials-back, previously seen images elicited 10% FP errors, while generally unseen images only produced 1.5% FP errors, as shown in Figure 3B. Since each of the two monkeys was tested for 30 catch trials per distance back, every point in Figure 3B has a sample size of ~60, sufficient for statistical analysis.

### Estimating rate of forgetting

**Figure 4:**
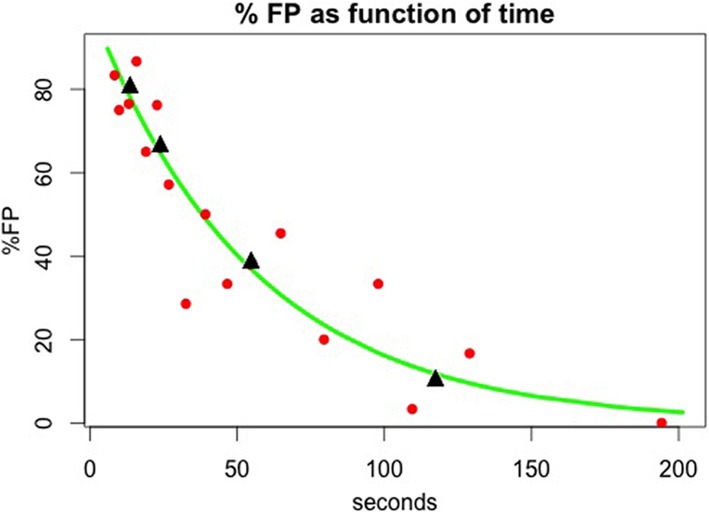
**Memory decay in the case where no reset mechanism has been activated.** The raw false positive (FP) and correct rejection (CR) data were fit to time between first image presentation and its repetition using a binomial generalized linear model with logarithmic link function yielding *FP* = exp (−0.18 * *t*), (*t* in seconds) or a decay factor of about 1/3 per min. Red dots correspond to FP rate and time average for data in exponentially increasing time intervals. Black triangles correspond to FP rate and time average over the data in 1, 2, 5, and 10 trials back. Neither of these averages was used for the fitting calculation.

The NST group never experienced repeated images from previous trials during training for Experiment I. We suggest that they did not develop an inter-trial reset mechanism. This gives us the unique opportunity to estimate natural memory decay for viewed images without reset. In Figure 4 we plot FP Rate (%) as a function of time between first (prior) presentation of the image and catch-trial presentation. The green line shows the result of fitting a binomial generalized linear model with logarithmic link function to the raw data of FPs and correct rejections as a function of time. This yields the fit *FP* = exp (−0.18 * *t*), (where FP is the False Positive rate and t is in seconds). This corresponds to a decay factor of about 1/3 per min. In Figure 4, each red dot represents average time and FP rate for data in exponentially increasing time intervals and the black triangles represent the averages for the data at each of the 1, 2, 5, and 10 back positions. *Y*-axis values are the same as in Figure 3B, but the *X*-axis represents physical time (instead of number of trials back). Note that black triangle or red dot average data were not used for the fitting calculation. We find that visual memory in monkeys gradually decreases, so that during this multiple-item discrimination task, even 10 trials later (almost 2 min, on average), about 10% of the on-average 44 images presented remain stored in memory and produce FP errors—more than five times the spontaneous FP rate!

### Experiment II

In Experiment II, monkey behavior for the DMMS task was tested by switching stimulus sets between monkey groups. Thus, Group FST switched to the unlimited novel image set (used by Group NST in Experiment I), and Group NST switched to the fixed image set used by Group FST in Experiment I. There was no adaptive pre-training for either group.

#### Group FST

**Figure 5:**
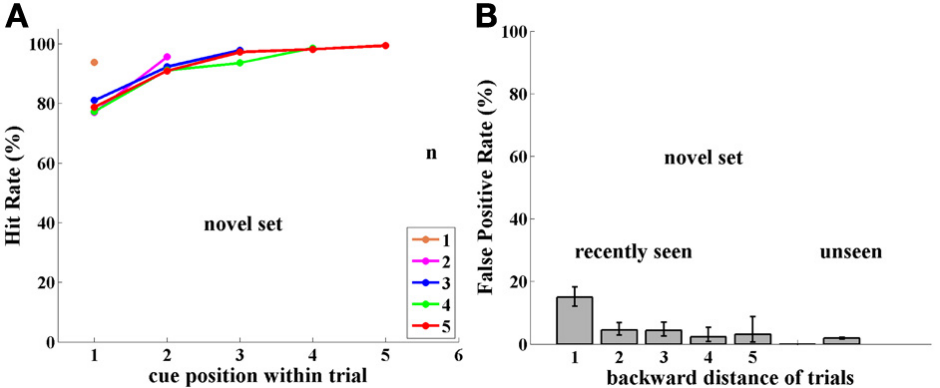
**Experiment II, Group FST, now tested with novel images. (A)** Performance (Hit Rate %); note similarity to Figure 3(A) for group NST. **(B)** FP rate for Group FST with novel images; Note similarity to FPs of same FST group with a fixed image set in Figure 2(B) and, surprisingly, not to the FPs of the NST group with novel images, shown in Figure 3(B).

***Performance.*** Performance of monkeys B and T with the unlimited set of novel images (over 4,751 trials) was significantly improved compared to their performance (in Experiment I) with the small fixed set of images (*p* < 0.001, for group and for each monkey separately). Performance showed significant improvement (*p* < 0.05) except when the cue immediately preceded the match. Performance was similar to that of Group NST in Experiment I (Figure 3A) and was 90% or greater, except when the cue was at the very beginning of the trial, as demonstrated in Figure 5A.

***False positives.*** We asked if repeated stimuli would elicit FP responses here, as well, as they did when monkeys D and L were initially tested with novel images, without prior experience with a fixed set of images. To test this, we introduced “catch trials” in 30% of trials and the repeated image could have been seen previously 1, 2, 3, 4, and 5 trials back. As before, each repeated image was shown as a “catch trial” only once, that is, each image could be shown a maximum of three times: as cue and match in its n-back-trial, and as sample in the “catch trial.”

Novel images seen in previous trials provoked FP errors mainly from the 1-back adjacent trial, as shown in Figure 5B, similar to the behavior with the fixed set of images (Figure 2B). Overall, novel images seen in previous trials provoked a FP rate of 7% for backward distances up to 5, compared with only 5% for Experiment I. Over the 5 backward distances, only the first was significantly higher for Experiment II at the 0.05 level, (*p*-values = 0.001, 0.38, 0.22, 0.84, 0.99; with similar values for each monkey separately). This is dramatically different from the result for Group NST in Experiment I, where they showed ~80% FPs. We conclude that the previous experience of this group with a fixed set allowed not only the possibility of avoiding FPs for the fixed set trial type, but also allowed such avoidance for the novel set; (see Discussion).

#### Group NST

**Figure 6:**
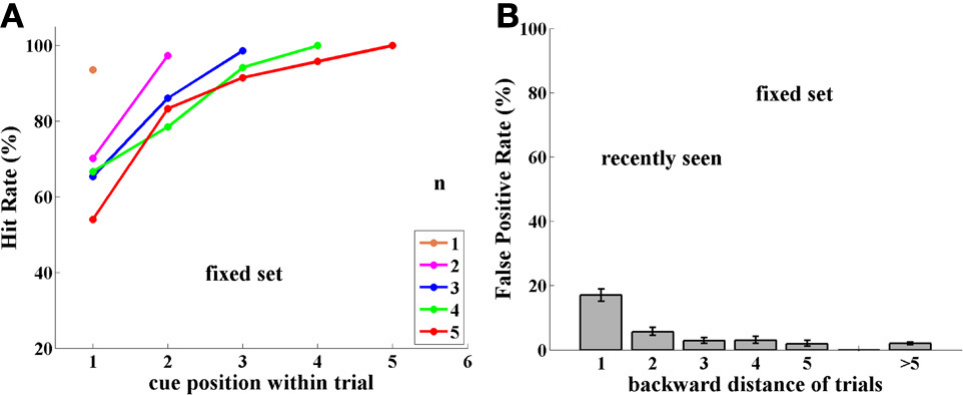
**Experiment II, Group NST, now tested with a fixed set of 16 images. (A)** Performance (Hit rate %). **(B)** FP rate. Note similarity to group FST shown in Figure 2.

***Performance.*** Switching from novel images to a small fixed set of images significantly impaired performance in monkeys D and L (3,680 trials; *p* < 0.001; for group and monkey L; *p* < 0.005 for monkey D)—as already seen in Experiment I in a cross-subject analysis. Again, as shown in Figure 6A, considering each fixed trial length, one sees that the more intervening images there were between cue and match, the more difficult it was to hold the cue image in memory. Note that when cue and match images were adjacent, performance is close to perfect—for all trial lengths. Overall, Group NST performance with the small fixed set of images in Experiment II is very similar to performance of Group FST in Experiment I with the same fixed set size (see Figure 2A).

***False positives.*** Switching from the unlimited set of novel images to the small fixed set dramatically influenced also the FP error rate in monkeys D and L. Images seen in previous trials elicited FP errors mostly when they were presented in the just preceding 1-back trial and to a small extent from 2-trials-back, as shown in Figure 6B. Over the 5 backward distances, only the first two were significantly higher for Experiment II at the.05 level, (*p*-values = 0.001, 0.003, 0.51, 0.86, 0.43; with similar values for each monkey separately). Thus, the FP error distribution for Group NST in Experiment II is rather similar to that for Group FST in Experiment I (compare Figures 2B, 6B). This reduction in FPs occurred very quickly, presumably when the monkeys noticed the frequently repeated images and their lack of producing reward.

### FP rate dependence on reward

We asked if the rate of FP errors depends on whether the trial where the image originally appeared was rewarded or unrewarded and whether it matters if the image was seen there once (as non-cue sample) or twice (as cue and match). That is: Does reward allow trial images to stay in memory, i.e., does reward hinder forgetting, and thus, increase FP errors? Is the memory strengthened by having seen the image twice? To estimate how reward and double viewing influence FP errors, we analyzed data from the experiments with the novel images, where there were many FPs. (FP data for the cases with fixed image sets are presented in the Supplementary Material; no significant differences were found between groups or between previous presentation rewarded vs. unrewarded).

**Figure 7:**
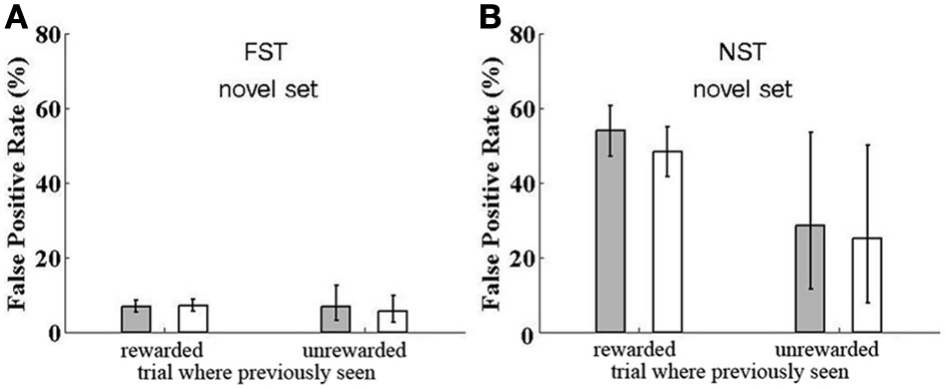
**Reward Dependence of False Positive rate for catch trials.** False Positive rate is shown for catch trials where a previously seen image is repeated, although the monkeys are expecting all images to be novel, except a single within-trial match repetition, to which they should respond. The FP rate is shown for trials where the catch image was previously presented in a trial which led to a correct response (rewarded) or not (unrewarded) and for trials where the twice-seen cue and match image is the one used for a catch trial (filled bar) or a once-seen sample was repeated in the catch trial (open bar). **(A)** Data for Group FST. **(B)** Data for Group NST. For both groups, data are shown for testing with novel images, except the catch trials.

For Group FST, who were initially trained with a fixed set of images and only subsequently tested with novel images, we saw above that the reset mechanism was transferred from the fixed set task to the novel images task. Here we found no dependence on reward in the previous trial. FP errors were found in 7% of catch trials, as shown in Figure 7A. This rate was the same for catch trials repeating an image from a trial which was successfully executed and the monkey received a reward, or from of a trial where the monkey missed the match or responded to a FP, and was not rewarded. It was also the same for catch trials repeating images that were presented as a sample (seen once), or images that were presented as a cue and then as a match (seen twice and rewarded, for recognizing match to cue of this image, or unrewarded, due to missing this match).

Results were very different for Group NST, who had no previous experience with the fixed set; they started out with the novel-images task and had no reset mechanism. Here, FP rate was highly affected by reward. As demonstrated in Figure 7B, catch trials with images from previously rewarded trials provoked 53% FPs if they were twice-seen cue and match images and 48% FPs if they were once-seen sample images. This difference is not significant (one-tail *t*-test by participant shows *p* = 0.34). Looking at the cases where the previous trial was unrewarded, we find provoked FP rates of only 29 (for twice-seen but match missed) and 25% (once-seen and trial unrewarded due to FP or Miss of another image). Again, the difference between twice- and once-seen is not significant (*p* = 0.50), nor is the difference between twice-seen and once-seen significant when considering both rewarded and unrewarded data (*p* = 0.40). However, the difference between rewarded and unrewarded cases (Figure 7B left vs. right) is indeed significant (*p* < 0.05). We conclude that when there is no reset mechanism available, the strength of image memory depends strongly on whether the trial in which the image appeared was rewarded. Reward strengthens the memory trace or synaptic strength enhancement. We return to this point in the Discussion.

## Discussion

We found that learning history has a great influence on how items are handled in monkey memory. Two groups learned the same paradigm, with their task being to watch a sequence of images and report repetition of any of the images within a trial. The only difference was that one group experienced this task with a fixed small set of 16 images, while the second group responded to an unlimited set of images, that is, every trial was composed of images never before shown to the monkey. The first group consistently saw the same images, trial after trial, with trials composed of images randomly selected from the fixed small set of 16 images. This was a challenge for memory and for forgetting because the paradigm required reporting every repetition of any image within the current trial, while ignoring the very same images seen very recently in previous trials. In some cases, when the current trial had as many as 6 samples, the time between cue and test was longer than that between a sample in the beginning of the trial and the (perhaps matching) image at the end of the preceding trial, a “repetition” the monkey was supposed to ignore.

Note that performance for experiments with a series of items followed by a single test item (present or absent from the series) displays both a recency and a primacy effect and a U-shape (Wright et al., 1985; Wright, 1998, 2007). The primacy effect is absent in our case, where attention and a decision must be made for each presented image, to determine if it matches any of the preceding images.

We suggest that to solve the problem of propagation of the memory of previously seen images to the following trial(s), the brain sets up an adaptive reset mechanism. The theory behind such a reset and a model describing its action are presented in an accompanying paper (Amit et al., 2013). In that paper we also integrate the model for familiarity recognition of novel images (Romani et al., 2008; Yakovlev et al., 2008), in the same network that handles repetition detection for well-learned images with Working Memory delay activity. Repetition detection with novel images relies on a small synaptic trace of the once-seen image, whereas with trained images it relies on their retention in Working Memory. These two types of detection entail different types of readout mechanisms. We propose that the reset mechanism, once acquired, can be applied to the novel image setting, though it requires an increased depression rate to sufficiently reduce the *synaptic* trace of once-seen images.

Note that it is not the presence of the match that initiates the reset, but rather the end of the trial (indicated by motor response; cessation of stimulus series; inter-trial interval). A physiological associate of this event was recorded as a burst of neuronal activity in a sample of pF neurons (Yakovlev and Hochstein, 2009). An alternative to the view that reset is the equivalent of directed forgetting is that somehow the monkey remembers the image but also remembers that it did not occur in the current trial and therefore, should not respond to its reappearance. We are not aware of physiological recordings supporting this alternative. Furthermore, this model does not explain the difference between 1-back and further back images.

Several primate neurophysiological studies demonstrated reduced neuronal responses when images are presented a second time within the same trial (as a match)—called “match suppression” (Brown et al., 1987; Riches et al., 1991; Li et al., 1993; Miller et al., 1993, 1996). On the other hand, despite suppression for within-trial repetitions, the response to an image's first appearance in each new trial is the same as to its original presentation (Hölscher and Rolls, 2002). This led to the conclusion that “perirhinal cortex neurons were actively reset between trials” (Hölscher and Rolls, 2002). Very recently it was observed that inhibitory IT neurons differentiate between novel and well-trained images (Woloszyn and Sheinberg, 2012), a signal that may be used for establishing a reset mechanism.

Previous human and animal studies have found a difference between multi-item memory for small vs. large stimulus set sizes (Mishkin and Delacour, 1975; Sands and Wright, 1980; Eacott et al., 1994; Wright, 2007; Basile and Hampton, 2010; Bigelow and Poremba, 2013). Performance with small sets is degraded due to what they call proactive interference, leading to FP responses. These reports do not directly discuss reset as the mechanism for overcoming such interference, and they do not use catch trials to determine the absence of reset for large set sizes—where it is not required.

Information stored in memory decays over time, but it is still unknown whether this is merely as a result of the passage of time or as a result of interference from new events (Wilson and Kipp, 1998; Lewandowsky and Oberauer, 2008; Hardt et al., 2013). Monkeys NST in experiment I were trained with no experience of repeated images from previous trials, hence they had not developed a reset mechanism. This presented the opportunity to estimate natural memory decay for seen images in a situation without reset. We found that gradual decrease of memory was best fit by an exponential function (Figure 4), consistent with previous reports (Sperling, 1960; Ringo, 1991; Jolicoeur, 1999; Fusi et al., 2007; Graziano and Sigman, 2008; Zylberberg et al., 2009; Bernacchia et al., 2011; Huang and Amit, 2011; Akrami et al., 2012). In our experiment, with monkeys performing a multiple-item discrimination task (the DMMS task), behavioral performance suggests that about 10% of images are still in memory after 2 min. In contrast it has been found that neurons in monkey anterior temporal lobe store information for seen images perhaps as long as 24 h (Xiang and Brown, 1998).

Reward significantly improves memory (Wittmann et al., 2005, 2008; Adcock et al., 2006; Bunzeck et al., 2012). In our experiment, monkeys from group FST showed no difference in the low rate of FP errors when the previous trial with these images were rewarded or not. Any reward effect might be marginal for the fixed set of well-learned images. We suggest it is not present in Experiment II of group FST because they had developed a reset mechanism, which presumably overwhelms the small effect of enhanced learning due to reward. Group FST were exposed to the novel images after developing the reset mechanism during sessions with a fixed set of images, so that the effect of enhanced learning due to reward cannot be observed. On the other hand, group NST monkeys, which had no reset mechanism during their first experiment with novel images, were significantly affected by reward. This seems to indicate that reward boosts image learning, perhaps through faster or greater synaptic potentiation. Thus, image meaning, behavioral response, or reward acquisition, enhances memory, though the effect is only observed for novel images.

We noted in the Results that performance was always poorer when the cue was the very first sample at the beginning of the trial, except when the Match immediately followed. Physiological studies in our lab found that there is delay activity in both IT and pF cortex. These have very different characteristics, however, in the context of multi-image memory tasks. IT delay activity is related to the last presented image, it is turned off by presentation of a new image, and it survives the inter-trial-interval (Yakovlev et al., 1998). pF delay activity survives presentation of a new image, so that multiple images can be actively remembered together (Miller et al., 1996), and, we now show, it is turned off at trial end. We propose that an interaction between the IT and pF representations leads to the anomalous results for cue position 1.

## Summary and conclusions

It has been found that multiple presentations of an image (such as the small fixed set used here), allow for development of delay-activity Working Memory. We propose that the visual system uses this type of memory mechanism to perform our DMMS task. This is also consistent with the performance decrease with Cue-to-Match distance, deriving from spontaneous forgetting of items, i.e., their dropping out of delay-activity. A major problem with performing this task is the need to remember only items of the current trial, and to forget—i.e., not to respond—to items seen recently, perhaps even in the 1-back trial. We propose a reset mechanism that turns off the delay activity between trials. This may be related to “directed forgetting,” a subject previously addressed without reference to specific underlying neural mechanisms (e.g., Bjork, 1972; Wilson and Kipp, 1998; Williams and Woodman, 2012; Williams et al., 2013), though in our case no explicit instruction is given to “forget” items from previous trials. For a recent review of active vs. passive forgetting, see (Hardt et al., 2013).

Performance with Novel images was surprisingly better than with the Fixed Set, and was independent of Cue-to-Match distance. We hypothesize (Yakovlev et al., 2008; Amit et al., 2013) that the same learning mechanism that builds synaptic strength to produce delay activity is also in operation here, though leaving a much weaker trace. Thus, the readout mechanism for detecting familiarity of once-seen images has to be different. For example, we predict that in this case monkeys will not learn associations, such as among sequential images (Sakai and Miyashita, 1991). Furthermore, since there were no repeated images (no catch trials) during training, there was no need to establish a reset mechanism. Indeed, when we introduced a small number of “catch” trials, we found a very large number of FP responses. Since there was no opportunity to learn to forget, the monkeys had a hard time forgetting.

Strikingly, when monkeys were trained first with a fixed set of images and established a reset mechanism, when they were switched to the novel images task, they were able to use this acquired reset mechanism for once-seen images as well. Catch trials induced only a limited number of FPs, mainly confined to images from the preceding trial. This finding suggests that reset is not just turning off delay activity—since there is no delay activity expected for novel images. Instead, we must conclude that reset also affects the synaptic structure initiated by seeing an image once, the synaptic structure that is responsible for a different response when a once-seen image is seen a second time.

Finally, we observed that monkeys without a reset mechanism easily remember dozens of once-seen images, with a fixed rate of decrease in repetition detection over time, and they are affected by the presence or absence of reward in previous trials.

### Conflict of interest statement

The authors declare that the research was conducted in the absence of any commercial or financial relationships that could be construed as a potential conflict of interest.
