# Supplementary material for: It's hard to forget: resetting memory in delay-match-to-multiple-image tasks
Source: Front Hum Neurosci. 2013 Nov 14;7:765. doi: 10.3389/fnhum.2013.00765 (PMC3827555; doi:10.3389/fnhum.2013.00765)
Supplement: Supplementary file 1 [file DataSheet2.PDF]

It's Hard to Forget: Resetting Memory in Delay-Match-to-Multiple-Image Tasks

Volodya Yakovlev, Yali Amit, and Shaul Hochstein

Supplementary Material

We present original data for all figures of the paper, computing for each the rates and Confidence Intervals (CI) of the data, including separate data for each monkey and figures for each monkey separately. Tables are numbered to correspond to those of the figures in the paper.

Table 2A. Group FST - Number of Hits, Misses, performance rate and Confidence Intervals (CI, at p<0.05 level) corresponding to data points in Figure 2A for group data, and figures below, for each monkey separately; q – cue position, n – trial length as number of samples, not including the match stimulus.

	B	T	B+T	B	T	B+T	B	T	B+T	B	T	B+T	B	T	B+T	B	T	B+T	
	q=1			q=2			q=3			q=4			q=5			q=6			
Hit	161	700	861																n=1
Miss	33	42	75																
Rate	83	94	92																
+CI	88	96	94																
-CI	77	92	90																
Hit	37	154	191	92	345	437													n=2
Miss	32	152	184	7	13	20													
Rate	54	50	51	93	96	96													
+CI	66	56	56	97	98	97													
-CI	41	45	46	86	94	93													
Hit	33	98	131	34	199	233	57	231	288										n=3
Miss	23	78	101	24	26	50	3	6	9										
Rate	59	56	56	59	88	82	95	97	97										
+CI	72	63	63	71	92	87	99	99	99										
-CI	45	48	50	45	84	77	86	95	94										
Hit	12	81	93	18	70	88	31	151	182	38	175	213							n=4
Miss	19	93	112	10	13	23	13	14	27	1	3	4							
Rate	39	47	45	64	84	79	70	92	87	97	98	98							
+CI	58	54	52	81	91	86	83	95	91	100	100	100							
-CI	22	39	38	44	75	71	55	86	82	87	95	95							
Hit	13	49	62	12	67	79	20	103	123	18	116	134	24	101	125				n=5
Miss	9	40	49	7	23	30	16	8	24	3	6	9	1	0	1				
Rate	59	55	56	63	74	72	56	93	84	86	95	94	96	100	99				
+CI	79	66	65	84	83	81	72	97	89	97	98	97	100	100	100				
-CI	36	44	46	38	64	63	38	86	77	64	90	88	80	96	95				
Hit	1	4	5	2	13	15	8	16	24	3	6	9	4	4	8	12	20	32	n=6
Miss	6	4	10	2	4	6	5	2	7	0	2	2	0	0	0	1	1	2	
Rate	14	50	33	50	76	71	62	89	77	100	75	82	100	100	100	92	95	94	
+CI	58	84	62	93	93	89	86	99	90	100	97	98	100	100	100	100	100	99	
-CI	0	16	12	7	50	48	32	65	59	29	35	48	40	40	63	64	76	80	

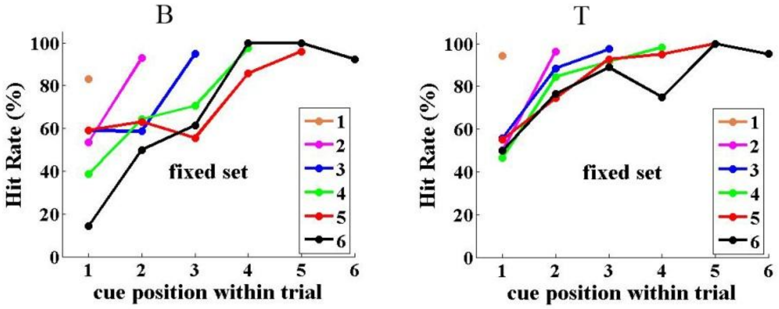

Table 2B. Group FST - False-positives (FPs), Correction-rejections (CRs), FP Rates and CIs (at p<0.05 level) corresponding to data points in Figure 2B (for group data, and figures below, for each monkey separately) for images previously seen, as function of number of trials before current trial where the same image was presented.

	Seen (no. of trials ago)																	
	1			2			3			4			5			> 5 ago		
	B	T	B+T	B	T	B+T	B	T	B+T	B	T	B+T	B	T	B+T	B	T	B+T
FPs	40	162	202	24	43	67	27	28	55	21	18	39	19	12	31	69	73	142
CRs	424	1,641	2,065	425	1,473	1,898	384	1,238	1,622	341	1,055	1,396	296	925	1,221	1,152	3,943	5,095
Rate	9	9	9	5	3	3	7	2	3	6	2	3	6	1	2	6	2	3
+CI	12	10	10	8	4	4	9	3	4	9	3	4	9	2	4	7	2	3
-CI	6	8	8	3	2	3	4	1	2	4	1	2	4	1	2	4	1	2

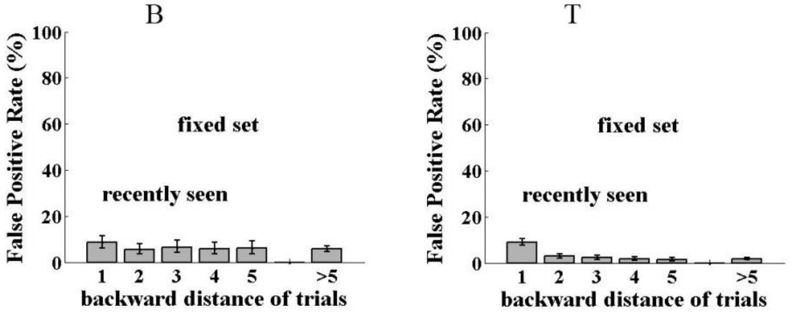

Table 3A. Group NST - Hits, Misses, performance rate and CIs corresponding to data in Figure 3A for group data, and figures below, for each monkey separately.

	D	L	D+L	D	L	D+L	D	L	D+L	D	L	D+L	D	L	D+L	
		q=1			q=2			q=3			q=4			q=5		
Hit	650	256	906													n=1
Miss	26	7	33													
Rate	96	97	96													
+CI	97	99	98													
-CI	94	95	95													
Hit	297	122	419	331	135	466										n=2
Miss	33	10	43	9	2	11										
Rate	90	92	91	97	99	98										
+CI	93	96	93	99	100	99										
-CI	86	87	88	95	95	96										
Hit	190	87	277	188	80	268	209	84	293							n=3
Miss	28	5	33	12	3	15	3	1	4							
Rate	87	95	89	94	96	95	99	99	99							
+CI	91	98	93	97	99	97	100	100	100							
-CI	82	88	85	90	90	91	96	94	97							
Hit	148	63	211	146	62	208	135	57	192	139	52	191				n=4
Miss	20	5	25	6	0	6	3	2	5	3	2	5				
Rate	88	93	89	96	100	97	98	97	97	98	96	97				
+CI	93	98	93	99	100	99	100	100	99	100	100	99				
-CI	82	84	85	92	94	94	94	88	94	94	87	94				
Hit	96	39	135	109	39	148	108	39	147	121	43	164	117	42	159	n=5
Miss	19	8	27	9	2	11	6	1	7	4	1	5	3	0	3	
Rate	83	83	83	92	95	93	95	98	95	97	98	97	98	100	98	
+CI	90	92	89	96	99	97	98	100	98	99	100	99	99	100	100	
-CI	75	69	77	86	83	88	89	87	91	92	88	93	93	92	95	

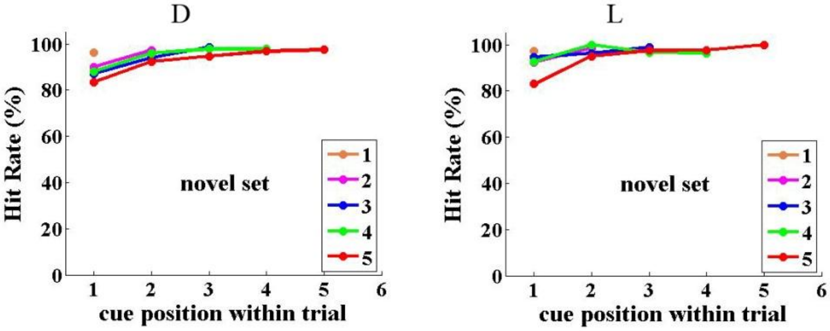

Table 3B. Group NST - FPs, CRs, FP Rates and CIs corresponding to data points in Figure 3B for group data, and figures below, for each monkey separately.

	Seen (no. of trials ago)														
		1			2			5			10			Unseen	
	D	L	D+L	D	L	D+L	D	L	D+L	D	L	D+L	D	L	D+L
FPs	27	22	49	19	20	39	16	7	23	3	3	6	176	34	210
CRs	2	10	12	9	11	20	15	22	37	28	25	53	9,708	3,805	13,513
Rate	93	69	80	68	65	66	52	24	38	10	11	10	2	1	2
+CI	99	84	89	84	81	78	70	44	52	26	28	21	2	1	2
-CI	77	50	68	48	45	53	33	10	26	2	2	4	0	0	1

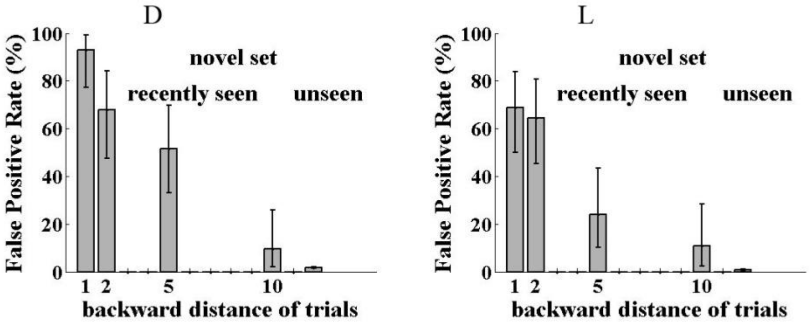

Table 5A. Group FST - Hits, Misses, performance rate and CIs for data in Figure 5A for group data, and figures below, for each monkey separately.

	B	T	B+T	B	T	B+T	B	T	B+T	B	T	B+T	B	T	B+T	
		q=1			q=2			q=3			q=4			q=5		
Hit	441	454	895													n=1
Miss	35	24	59													
Rate	93	95	94													
+CI	95	97	95													
-CI	90	93	92													
Hit	179	148	327	241	225	466										n=2
Miss	45	53	98	15	6	21										
Rate	80	74	77	94	97	96										
+CI	85	80	81	97	99	97										
-CI	74	67	73	91	94	93										
Hit	138	114	252	136	140	276	140	135	275							n=3
Miss	24	35	59	13	10	23	6	0	6							
Rate	85	77	81	91	93	92	96	100	98							
+CI	90	83	85	95	97	95	98	100	99							
-CI	79	69	76	86	88	89	91	97	95							
Hit	94	76	170	102	103	205	102	102	204	95	104	199				n=4
Miss	22	28	50	12	8	20	7	7	14	3	0	3				
Rate	81	73	77	89	93	91	94	94	94	97	100	99				
+CI	88	81	83	94	97	94	97	97	96	99	100	100				
-CI	73	63	71	82	86	87	87	87	89	91	97	96				
Hit	65	54	119	67	73	140	66	75	141	82	79	161	85	72	157	n=5
Miss	17	15	32	11	3	14	0	4	4	0	3	3	1	0	1	
Rate	79	78	79	86	96	91	100	95	97	100	96	98	99	100	99	
+CI	87	87	85	93	99	95	100	99	99	100	99	100	100	100	100	
-CI	69	67	71	76	89	85	95	88	93	96	90	95	94	95	97	

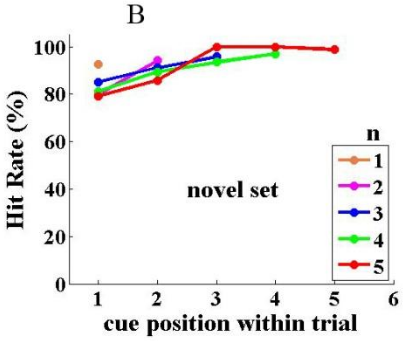

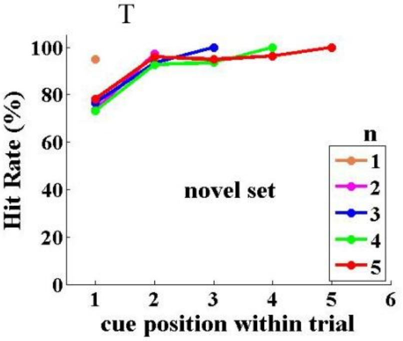

Table 5B. Group FST - FPs, CRs, FP Rates and CIs for data in Figure 5B for group data, and figures below, for each monkey separately.

	Seen (no. of trials ago)																	
		1			2			3			4			5			Unseen	
	B	T	B+T	B	T	B+T	B	T	B+T	B	T	B+T	B	T	B+T	B	T	B+T
FPs	34	46	80	5	16	21	5	11	16	3	2	5	3	0	3	130	102	232
CRs	158	296	454	157	281	438	117	234	351	152	59	211	94	0	94	6237	5825	12,062
Rate	18	13	15	3	5	5	4	4	4	2	3	2	3	na	3	2	2	2
+CI	24	18	18	7	9	7	9	8	7	6	11	5	9	na	9	2	2	2
-CI	13	10	12	1	3	3	1	2	3	0	0	1	1	na	1	2	1	2

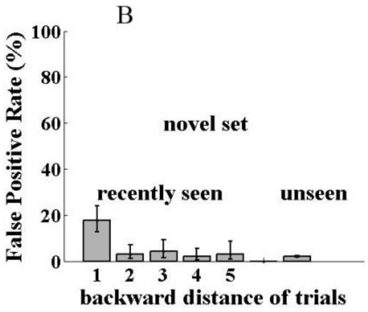

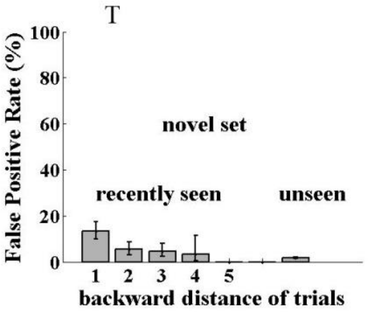

Table 6A. Group NST - Hits, Misses, performance rate and CIs for data in Figure 6A for group data, and figures below, for each monkey separately.

	D	L	D+L	D	L	D+L	D	L	D+L	D	L	D+L	D	L	D+L	
		q=1			q=2			q=3			q=4			q=5		
Hit	515	196	711													n=1
Miss	42	7	49													
Rate	92	97	94													
+CI	95	99	95													
-CI	90	93	92													
Hit	161	63	224	279	91	370										n=2
Miss	64	31	95	8	2	10										
Rate	72	67	70	97	98	97										
+CI	77	76	75	99	100	99										
-CI	65	57	65	95	92	95										
Hit	116	35	151	124	55	179	157	64	221							n=3
Miss	55	25	80	22	7	29	2	1	3							
Rate	68	58	65	85	89	86	99	98	99							
+CI	75	71	71	90	95	90	100	100	100							
-CI	60	45	59	78	78	81	96	92	96							
Hit	64	18	82	77	29	106	142	36	178	97	44	141				n=4
Miss	23	18	41	18	11	29	5	6	11	0	0	0				
Rate	74	50	67	81	73	79	97	86	94	100	100	100				
+CI	82	67	75	88	85	85	99	95	97	100	100	100				
-CI	63	33	58	72	56	71	92	71	90	96	92	97				
Hit	36	11	47	62	23	85	63	23	86	41	27	68	75	35	110	n=5
Miss	29	11	40	11	6	17	5	3	8	2	1	3	0	0	0	
Rate	55	50	54	85	79	83	93	88	91	95	96	96	100	100	100	
+CI	68	72	65	92	92	90	98	98	96	99	100	99	100	100	100	
-CI	43	28	43	75	60	75	84	70	84	84	82	88	95	90	97	

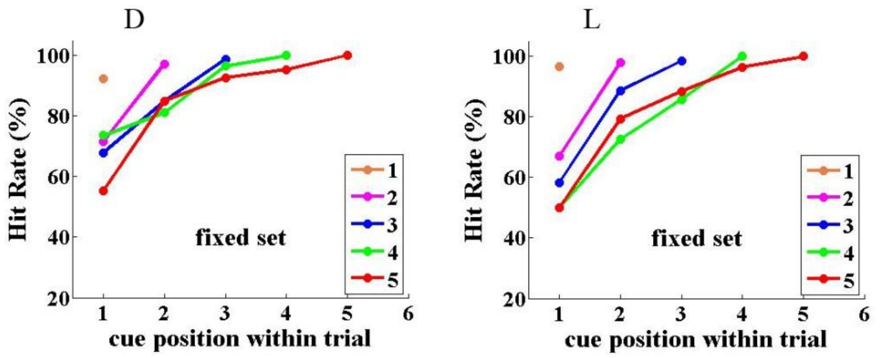

Table 6B. Group NST - FPs, CRs, FP Rates and CIs for data in Figure 6B for group data, and figures below, for each monkey separately.

	Seen (no. of trials ago)																	
		1			2			3			4			5			> 5 ago	
	D	L	D+L	D	L	D+L	D	L	D+L	D	L	D+L	D	L	D+L	D	L	D+L
FPs	239	18	257	62	16	78	27	9	36	15	14	29	11	7	18	57	31	88
CRs	1,146	108	1,254	1,106	207	1,313	938	316	1,254	607	357	964	524	412	936	2,963	1,360	4,323
Rate	17	14	17	5	7	6	3	3	3	2	4	3	2	2	2	2	2	2
+CI	19	22	19	7	11	7	4	5	4	4	6	4	4	3	3	2	3	2
-CI	15	9	15	4	4	4	2	1	2	1	2	2	1	1	1	1	2	2

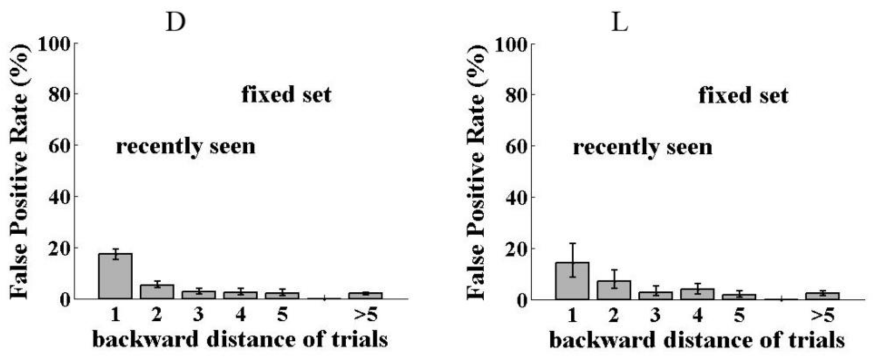

Table 7A. Group FST - Reward Dependence of FP rate for catch trials with novel images. FPs, CRs, FP Rates and CIs for data in Figure 7A.

	rewarded test	rewarded non-test	unrewarded test	unrewarded non-test
FPs	76	73	9	10
CRs	1036	961	125	173
Rate	6.83	7.06	6.72	5.46
+CI	8.48	8.8	12.37	9.82
-CI	5.42	5.57	3.12	2.65

Table 7B. Group NST - Reward Dependence of FP rate for catch trials with novel images. FPs, CRs, FP Rates and CIs for data in Figure 7B.

	rewarded test	rewarded non-test	unrewarded test	unrewarded non-test
FPs	55	57	2	3
CRs	47	61	5	9
Rate	53.92	48.31	28.57	25
+CI	63.84	57.69	70.96	57.19
-CI	43.77	39.01	3.67	5.49

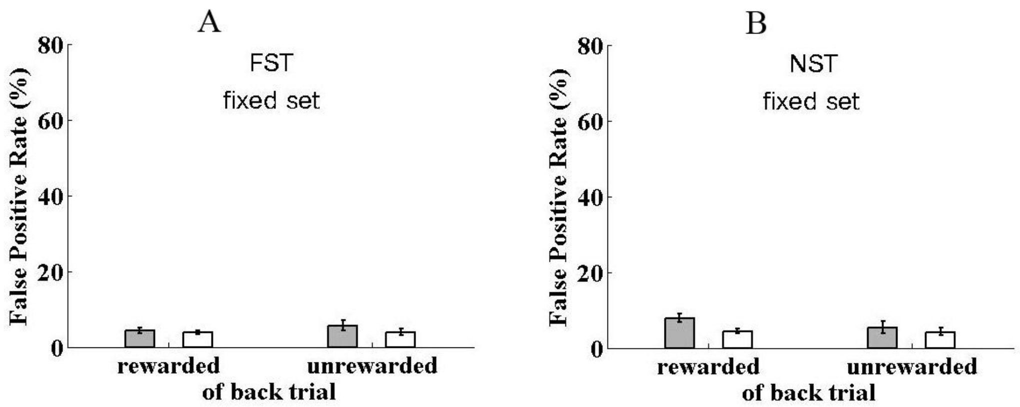

Figure S1. Reward Dependence of False Positive rate for catch trials for tests with a fixed set of 16 images. There are far lower FP rates here, as the monkeys establish a reset mechanism, "forgetting" the images seen in preceding trials, presumably by curtailing the delay activity. False Positive rate is shown for images which were previously seen, as a function of whether they were shown once (as a sample – white bar) or twice (as cue and match – black bar), and whether the trial was rewarded or unrewarded. Use of a fixed set of only 16 images means that there are very many cases of repeated images. A – data for Group FST, their first data. B – data for Group NST, who were previously trained with novel images.

Table S1A. Group FST - Reward Dependence of FP rate for catch trials with fixed set images. FPs, CRs, FP Rates and CIs for data in Figure S1A.

	rewarded seen twice	rewarded seen once	unrewarded seen twice	unrewarded seen once
FPs	125	209	61	103
CRs	2758	5334	1039	2535
Rate	4.34	3.77	5.55	3.9
+CI	5.14	4.31	7.07	4.72
-CI	3.62	3.28	4.27	3.2

Table S1B. Group NST - Reward Dependence of FP rate for catch trials with fixed set images. FPs, CRs, FP Rates and CIs for data in Figure S1B.

	rewarded seen twice	rewarded seen once	unrewarded seen twice	unrewarded seen once
FPs	189	184	43	75
CRs	2232	4084	769	1727
Rate	7.81	4.31	5.3	4.16
+CI	8.95	4.96	7.07	5.19
-CI	6.77	3.72	3.86	3.29
